# Interventions to improve patients’ compliance with therapies aimed at lowering glycated hemoglobin (HbA1c) in type 1 diabetes: systematic review and meta-analyses of randomized controlled clinical trials of psychological, telecare, and educational interventions

**DOI:** 10.1186/s13063-016-1207-6

**Published:** 2016-02-17

**Authors:** Luciana Verçoza Viana, Marilia Brito Gomes, Lenita Zajdenverg, Elizabeth Joao Pavin, Mirela Jobim Azevedo, On Behalf of the Brazilian Type 1 Diabetes Study Group (BrazDiab1SG)

**Affiliations:** Endocrinology Division, Hospital de Clínicas de Porto Alegre, Universidade Federal do Rio Grande do Sul, Rua Ramiro Barcelos 2350, Prédio 12, 4° andar, 90035-003 Porto Alegre, RS Brazil; Unit of Diabetes, Universidade Estadual do Rio de Janeiro, Rio de Janeiro, Brazil; Internal Medicine Department, Diabetes Division, Hospital Universitário Clementino Fraga Filho, Universidade Federal do Rio de Janeiro, Rio de Janeiro, Brazil; Department of Clinical Medicine, Universidade Estadual de Campinas, Campinas, Brazil

**Keywords:** Adherence, Non-pharmacological interventions, Type 1 diabetes, Systematic review, Meta-analyses

## Abstract

**Background:**

Brazilian records on glycemic control in patients with type 1 diabetes show treatment efficacy. Poor patient adherence to therapeutic proposals influences these results and can be associated with social, psychological, and economic aspects, besides others factors. The aim of this study was to evaluate the efficacy of psychological, telecare, and educational interventions to improve treatment compliance among patients with type 1 diabetes. Compliance was assessed indirectly using reduction of glycated hemoglobin (HbA1c) as the principal outcome measure.

**Methods:**

Systematic review and meta-analyses of randomized controlled clinical trials (RCTs) were performed using Medline, Embase, Cochrane and Scopus databases up to April 2015. The following medical subject headings were used: Diabetes Mellitus, Type 1, Patient Compliance or Adherence, Hemoglobin A, glycated, and Randomized Controlled Trial. The principal outcome was change in HbA1c between baseline and follow-up. Where appropriate, trials were combined in meta-analysis using fixed effects models.

**Results:**

From 191 articles initially identified, 57 were full text reviewed, and 19 articles met the inclusion criteria providing data from 1782 patients (49.4 % males, age 18 years). The RCTs (2 to 24 months in duration) were divided into four groups according to type of intervention: psychology (seven studies; 818 patients), telecare (six studies; 494 patients); education (five studies; 349 patients), and psychoeducation (one study; 153 patients). All studies reported some type of adherence measurement of the interventions. Decrease in HbA1c was observed after psychology (MD −0.310; 95 % CI, −0.599 to −0.0210, *P* = 0.035) but not after telecare (MD −0.124 %; 95 % CI, −0.268, 0.020; *P* = 0.090) or educational (MD −0.001; 95 % CI, −0.202, 0.200; *P* = 0.990) interventions.

**Conclusion:**

Psychological approaches to improve adherence to diabetes care treatment modestly reduced HbA1c in patients with type 1 diabetes; telecare and education interventions did not change glycemic control. However, the limited number of studies included as well as their methodological quality should be taken into account.

**Electronic supplementary material:**

The online version of this article (doi:10.1186/s13063-016-1207-6) contains supplementary material, which is available to authorized users.

## Background

A seminal study published in recent decades clearly demonstrated that intensive glycemic treatment promoting lower glycated hemoglobin (HbA1c) values, as compared to standard care, can prevent or postpone chronic diabetic complications [1]. Furthermore, follow-up of these patients after the end-of-studies demonstrated that past strict glycemic control was associated with a low prevalence of complications years later. Patients intensively treated early in the course of type 1 diabetes less frequently developed impaired glomerular filtration rate [2], increased urinary albumin excretion [2, 3], and also had a lower risk of cardiovascular disease [4] than those treated with conventional diabetes therapy. Reduction in the risk of cardiovascular, renal, and ocular disease by strict glycemic control was recently reinforced in a systematic review in these patients [5].

HbA1c measurement has been widely used to evaluate glycemic control in patients with diabetes. It reflects the average glycemia over several months [6] and should be measured every 3 months. Whenever possible HbA1c targets should be maintained as close as possible to the non-diabetic levels (<6.5 %) but goals must be individualized by age and by the presence of chronic diabetic complications [7]. Diabetes management requires adherence to a complex daily therapeutic regimen in order to reduce HbA1c levels. Patients have to be able to adhere to many procedures such as self-blood glucose monitoring, diet plan, insulin administration and dose titration, and exercise [6].

Although epidemiological data on patients with type 1 diabetes in Brazil are still scarce, incidence seems to be increasing (incidence rate of 18.49/100,000) [8]. Indeed, the direct medical costs of type 1 diabetes in Brazil are about US$1319.15 per patient for our national health service, not including the expenditure on chronic diabetic complications [9]. This aspect is relevant since the majority of our patients are at high risk of developing chronic diabetic complications. A survey conducted in 573 patients with type 1 diabetes in the south of Brazil demonstrated a high prevalence of diabetic retinopathy (43.3 %) and diabetic kidney disease (34.5 %) [10].

Despite advances in therapeutics, poor glycemic control is still a reality in many type 1 diabetic patients [11, 12]. Accordingly, up to 78 % of Brazilian patients with type 1 diabetes do not attain glycemic targets. Table 1 shows mean HbA1c and the percentage of patients with type 1 diabetes who attain glycemic targets in different Brazilians centers.

**Table 1:** Mean glycated hemoglobin (HbA1c) and percentage of patients with type 1 diabetes who are on glycemic target in Brazilian centers

Author	Number	Region of Brazil	HbA1c (%)	Percentage of patients on glycemic target*
			(mean ± SD)	
Rodrigues et al. 2010 [10]^a^	573	South	9.0 ± 3.9	22.0 %
Mendes et al. 2010 [49]^b^	979	South, Southeast, Northeast, Middle-west	-	7.0 %
Gomes et al. 2012 [50]^c^	3591	South, Southeast, North/Northeast, Middle-west	9.1 ± 2.3 to 9.4 ± 2.6	12.2 to 21.4 %
Gomes et al. 2012 [51]^d^	1774	South, Southeast, North/Northeast, Middle-west	9.1 ± 2.2	11.6 %
Viana et al. 2013 [52]^e^	1026	South, Southeast, North/Northeast, Middle-west	9.3 ± 2.3	13.0 %

*Glycemic targets: ^a, b, e^HbA1c <7.0 %; ^c, d^HbA1c <7 % – adults, HbA1c <7.5 % – 13 to 19 years, HbA1c < 8 % – 6 to 12 years, HbA1c >7.5 % and HbA1c <8.5 % – < 6 years

Poor compliance with diabetes treatment is probably an important determinant of poor glycemic control observed in patients with type 1 diabetes. As adherence to treatment increases, HbA1c decreases as demonstrated by a meta-analysis of 21 cross-sectional studies including 2492 youth with type 1 diabetes [13]. Multicomponent adherence or self-management promoting interventions seems to be more potent than single ones, although with a borderline beneficial effect on HbA1c [14]. Many factors have been associated with adherence to diabetes treatment and glycemic control such as economic status [15], access to diabetes care [16] and devices to self-monitor blood glucose [17], family support [18], social and peer pressures [19], interactions with their health-care providers [16], presence of depression [18], and transition to adolescence [18–20]. In this sense, non-pharmacological strategies for improving adherence to diabetes care, resulting in improved glycemic control, have been studied: psychological [21–29], telecare or Internet-based [30–35], educational [26, 36–38], and psychoeducational [39] interventions. Although other meta-analyses [30, 40], have already examined the effect of non-pharmacological interventions on compliance with diabetes treatment, the efficacy of such strategies is still uncertain.

Considering the poor glycemic control, the high prevalence of chronic diabetic complications, and the increasing worldwide prevalence of type 1 diabetes among children and adolescents [41, 42] it is crucial to identify factors that improve adherence to diabetes treatment. The aim of this study was to evaluate the efficacy of psychological, telecare, and educational interventions to improve treatment compliance among patients with type 1 diabetes. Compliance was assessed indirectly using reduction of HbA1c as the principal outcome measure.

## Methods

This systematic review was carried out using a protocol constructed according to the Cochrane Handbook recommendations [43] and reported in accordance with Preferred Reporting Items for Systematic Reviews and Meta-Analyses (PRISMA) statement [44] (Additional file 1).

### Data sources and searches

We searched databases from Medline, Embase, Cochrane, and Scopus to identify randomized controlled clinical trials (RCTs) that reported non-pharmacological interventions to improve adherence to diabetes treatment in patients with type 1 diabetes up to April 2015. The initial search comprised the following medical subject headings: *“Diabetes Mellitus, Type 1” [Mesh], “Patient Compliance” [Mesh], or Adherence*, *“Hemoglobin A, Glycated” [Mesh],* and related entry terms associated with a high sensitivity strategy for the search of RCTs available at http://www.sign.ac.uk/methodology/filters.html#random (see Appendix section). All potentially eligible studies were considered for review, limited to the English, Spanish, or Portuguese language. A manual search was also performed in the reference lists of included articles.

### Study selection

We included RCTs that reported changes in the HbA1c as differences between final and baseline interventions in RCTs. We excluded studies if they were not randomized, were crossover trials, included patients with type 1 and type 2 diabetes being analyzed together, included pregnant patients, or had no information about HbA1c.

### Data extraction and quality assessment

All citations retrieved from electronic databases were imported to the EndNote Program. Two reviewers (MJA, LVV) independently analyzed the titles and abstracts of every paper retrieved from the literature search to identify potentially eligible studies. All studies that did not meet the inclusion criteria were excluded. The full text of the remaining papers was obtained for further examination. The same two reviewers using a standardized data extraction form independently extracted data of the included studies. Extracted data included first author’s name, year of publication, number of participants, details of the study design (i.e., randomization method), trial duration, and patient characteristics (age, gender, ethnicity, diabetes duration). Studies were divided into four categories according to the type of intervention: psychology, telecare, education, and psychoeducation. Briefly, telecare intervention was defined as teleconsultation, tele-expertise, or telemonitoring [45]. Behavioral, multisystemic, and motivational approaches were considered a psychological intervention and any structured educational program as an educational intervention. Psychoeducation intervention was defined when psychological and educational tools were implemented at the same intervention.

Methodological quality assessment of included RCTs was independently assessed by the same two reviewers (MJA, LVV). We used the Cochrane Collaboration tool for assessing risk of bias of every included study. According to the Cochrane Collaboration, biases were classified into six domains: selection, performance, detection, attrition, reporting, and other [43, 46]. The risk of bias for each domain was classified as high, low, or unclear.

### Data synthesis and analysis

Descriptive data from the systematic review were presented as mean and/or range, when available. We analyzed HbA1c (%) as a continuous variable and reported HbA1c changes as absolute differences between arithmetic means at baseline and end-of-study and mean differences (MD) were used in the analyses (fixed models).

The heterogeneity between the studies was evaluated by Cochran’s chi-squared test (*Q* test) and a *P* value for trend ≤0.10 was considered statistically significant. The *I*^*2*^ test was also performed to evaluate the magnitude of heterogeneity [47] and statistical heterogeneity was considered in the presence of *I*^*2*^ values >75 %. Subgroup analyses were performed including only RCTs conducted with children and teenagers.

All statistical analyses will be performed using Stata 11.0 software (StataCorp, College Station, TX, USA). Significance was set at *P* <0.05 and 95 % confidence intervals are quoted throughout.

## Results

### Literature search

**Fig. 1:**
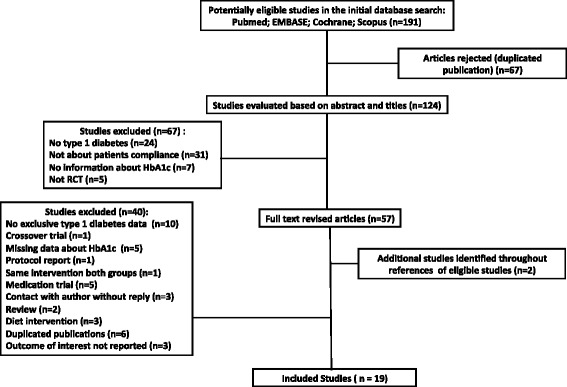
Flow diagram of literature search to identify randomized clinical trials evaluating interventions to improve compliance with lower glycated hemoglobin (HbA1c) values in patients with type 1 diabetes

We identified 191 studies in database searches. Of these, 67 studies were excluded due to duplication. Another 67 articles were excluded based on title or abstract: 24 studies were not performed in patients with type 1 diabetes; 31 studies had no information about treatment compliance; seven studies did not report HbA1c; and five studies had a non-randomized design. Then we evaluated the full texts of 57 articles. Two additional papers identified in the references of the revised articles were also fully evaluated. Hence, from a total of 57 studies, 40 were excluded and 19 trials, which fulfilled all selection criteria, were included in the current systematic review (Fig. 1).

### Study characteristics

This systematic review included a total of 1782 patients with type 1 diabetes aged 18 years (12 to 46), 49.4 % males, 71.6 % of white ethnicity, and with a mean duration of diabetes of 9.3 years (3.7 to 23). Baseline mean HbA1c in intervention groups ranged from 8.2 % to 11.4 % and from 8.2 % to 11.3 % in the control groups. Trial duration varied from 2 to 24 months.

RCT characteristics according to each intervention category are described in Table 2. Table 3 depicts risk of bias in each individual RCT evaluating interventions to improve compliance with lower HbA1c in patients with type 1 diabetes. Most of the quality domains of studies included in these meta-analyses revealed a low or uncertain bias risk.

**Table 2:** Characteristics of included randomized clinical trials evaluating interventions to improve compliance with lower glycated hemoglobin (HbA1c) in patients with type 1 diabetes according to intervention categories

Study	Sample	Intervention and Control groups	HbA1c changes/comments
Psychological category			
Ellis, 2005, 2007 [21–24] (4 published complementary reports)	*n* = 127 Age = 13.3 years Diabetes duration = 5.3 years Male = 62 (48 %) White = 33 (26 %)	Intervention Multisystemic therapy: intensive and home- and community-based, originally designed for youths with antisocial behavior. Duration of intervention: 5.7 months Control Standard medical care: quarterly visit of multidisciplinary team	Intervention Baseline = 11.4 ± 2.2 % End-of-study = 10.8 ± 2.6 % Control Baseline = 11.3 ± 2.3 % End-of-study = 11.3 ± 2.3 % Significant reduction of HbA1c only in the intervention group Compliance evaluation of the psychological intervention: semi structured interview
Nansel, 2007 [25]	*n* = 81 Age =13.6 years Diabetes duration = 7.6 years Male = 36 (44 %) White = 69 (85 %)	Intervention “Diabetes Personal Trainer”: approach guided by principles of motivational interviewing, applied behavior analysis, and problem solving. Duration of intervention: 2 months Control Education plus standard diabetes care	No significant reduction in HbA1c in intervention and control groups, but absolute values were not described Compliance evaluation of the psychological intervention: modified version of Diabetes –Management Profile
Weinger, 2011 [26]	*n* = 110^a^ Age = 46.6 years Diabetes duration = 23.7 years Male = 48 (48 %) White = 105 (96 %)	Intervention Structured behavioral intervention: five 2-hour sessions. Duration of intervention: 6 weeks Control Individual appointments with diabetes nurse and dietitian educators	Intervention Baseline = 9.0 ± 1.9 % End-of-study = 8.7 ± 1.2 % Control Baseline = 8.7 ± 0.6 % End-of-study = 8.5 ± 1.1 % Changes of HbA1c were described but statistical analysis was not reported Compliance evaluation of the psychological intervention: frequency of diabetes self-care, 3-day pedometer readings, 24-hour diet recalls, average number of glucose checks.
Nansel, 2012 [27]	*n* = 390 Age = 12.5 years Diabetes duration = 4.8 years Male = 191 (49 %) White = 273 (70 %)	Intervention Clinic-integrated behavioral: designed to improve family diabetes management (WE-CAN manage diabetes). Duration of intervention: 24 months Control Standard medical care	Significant reduction of HbA1c occurred only in the intervention group, but absolute values were not described Compliance evaluation of the psychological intervention: semi structured interview
Mulvaney, 2010 [28]	*n* = 72 Age =15.1 years Diabetes duration: 6.3 years Male = 40 (56 %) White = 66 (92 %)	Intervention Learning, social-cognitive and self-determination management by website support. Duration of intervention: 11 weeks. Control Usual care	Intervention Baseline = 9.1 ± 1.9 % End-of –study = 9.1 ± 1.8 % Control Baseline = 8.2 ± 1.2 % End-of-study = 8.5 ± 1.3 % No significant reduction in HbA1c in intervention and control groups Compliance evaluation of the psychological intervention: The Diabetes Rating Scale
Franklin, 2006 [29]	*n* = 61 Age = 13.5 years Diabetes duration = 4.1 years Male = 34 (56 %) White = 59 (97 %) Results referred only to patients on conventional insulin arm	Intervention “Sweet talk”: motivational support network to deliver behavioral intervention through mobile. Duration of intervention: unclear Control Usual care	Intervention Baseline = 9.8 % End-of-study = 10.1 ± 1.7 % Control Baseline = 10.1 % End-of-study = 10.3 ± 1.7 % No significant reduction in HbA1c in intervention and control groups Compliance evaluation of the psychological intervention: self-report adherence
Telecare category			
Montori, 2004 [30]	*n* = 31 Age = 43 years Diabetes duration = 17 years Male = 10 (32 %) White = no information	Intervention Monitoring blood glucose four times/day and transmitting recorded data twice a week with feedback from a nurse supervised by an endocrinologist 24 hours after the transmission. Duration of intervention: 6 months Control Same monitoring requested but without feedback	Intervention Baseline = 9.3 ± 1.3 % End-of-study = 7.8 ± 1.3 % Control Baseline = 8.8 ± 1.2 % End-of-study = 8.2 ± 1.2 % Significant reduction of HbA1c only in the intervention group Compliance evaluation of the telecare intervention: SMBG and insulin use
Lawson, 2005 [31]	*n* = 46 Age = 15.2 years Diabetes duration = 6.5 years Male = 26 (56 %) White = no information	Intervention Weekly standardized telephone contact with a diabetic nurse specialist to discuss blood sugar over the last week and performing insulin adjustments using standard rules and algorithms. Duration of intervention: 6 months Control Standard care with quarterly visit with a nurse and an endocrinologist	Intervention Baseline = 10 ± 1.3 % End-of-study = 9.4 ± 1.4 % Control Baseline = 9.7 ± 0 .6 % End-of-study = 9.2 ± 1.4 % No significant reduction of HbA1c in intervention and control groups Compliance evaluation of the telecare intervention: general adherence with diabetes management (blood glucose testing, insulin schedule, food plan, glucose goals, exercise)
Farmer, 2005 [32]	*n* = 93 Age = 23.8 years Diabetes duration = 12.1 years Male = 55 (59 %) White = no information	Intervention Clinical advice and structured specialized nurse counseling in response to real-time blood glucose test results. Duration of intervention: 9 months Control Data transmission without feedback	Intervention Baseline = 9.2 ± 1.1 % End-of-study = 8.6 ± 1.4 % Control Baseline = 9.3 ± 1.5 % End-of-study = 8.9 ± 1.4 % Significant reduction of HbA1c in intervention and control groups, without difference between them Compliance evaluation of the telecare intervention: SMBG
Landau, 2011 [33]	*n* = 70 Age = 15 years Diabetes duration = 5.7 years Male = 32 (46 %) White = no information	Intervention Weekly upload of the self-monitoring blood glucose and feedback from study coordinator. Parents were contacted if any change in the treatment was necessary. Duration of intervention: 6 months Control Data upload without study coordinator feedback	Intervention Baseline = 8.5 ± 1.4 % End-of-study = 8.5 ± 1.4 Control Baseline = 8.2 ± 1.1 % End-of-study = 8.4 ± 1.1 % No significant reduction of HbA1c in intervention and control groups Compliance evaluation of the telecare intervention: SMBG
Gay, 2006 [34]	*n* = 100 Age = 13.3 years Diabetes duration = 6.2 years Male = 32 (61 %) White = no information	Intervention Twice a month children went to a selected pharmacy to download data stored in their glucometer. Data was transmitted to a pediatric diabetologist and within 5 days feedback was provided. Duration of intervention: 6 months Control Usual follow-up	Intervention Baseline = 9.2 ± 1.1 % End-of-study = 9.1 ± 1.5 % Control Baseline = 9.2 ± 1 % End-of-study = 9.3 ± 1.2 % No significant reduction of HbA1c in intervention and control groups. There were problems with software installation Compliance evaluation of the telecare intervention: SMBG and insulin adjustments
Esmatjes, 2014 [35]	*n* = 154 Age = 31.7 years Diabetes duration = 17.7 years Male = 69 (44.9 %) White = no information	Intervention Five telematic visits, and management of the Medical Guard Diabetes (MGD) system (Pulso Ediciones, Barcelona, Spain) with data reports once a month and responses of diabetes team in the following 3 days with recommendations on treatment adjustments. Duration of intervention: 6 months Control All visits were in hospital and data were obtained on site during the visits	Intervention Baseline = 9.3 ± 1.5 % End-of-study = 8.7 ± 1.5 % Control Baseline = 9.2 ± 0.9 % End-of-study = 8.6 ± 0.9 % No significant reduction of HbA1c between intervention and control groups Compliance evaluation of the telecare intervention: self-care treatment adherence
Educational category			
Cook, 2002 [36]	*n* = 53 Age = 14.6 years Diabetes duration = no information Male = 26 (49 %) White = 45 (85 %)	Intervention Small group education to teach adolescents to became more responsible with day-to-day diabetes care (Choices Program). Duration of intervention: 6 weeks Control Usual care	Intervention Baseline = 8.9 ± 1.3 % End-of-study = 8.3 ± 1.4 % Control Baseline = 9.3 ± 2 .1 % End-of-study = 9.0 ± 1.9 % No significant reduction of HbA1c in intervention and control groups at 6 months Compliance evaluation of the educational intervention: SMBG and Diabetes Problem Solving Questionnaire
Howe, 2005 [37]	*n* = 49 Age = 12.8 years Diabetes duration = no information Male = 28 (57 %) White = 27 (55 %)	Intervention Single educational intervention to provide families with basic diabetes management skills. Duration of intervention: one session Control Standard care with quarterly visit with a nurse practitioner and an endocrinologist	Intervention Baseline = 10.1 ± 1.2 % End-of-study = 9.7 ± 1.9 % Control Baseline = 10.2 ± 1.4 % End-of-study = 9.9 ± 1.6 % No significant reduction of HbA1c in intervention and control groups Compliance evaluation of the educational intervention: Adherence Clinician Checklist
Howe, 2005 [37]	*n* = 54 Age = 12.1 years Diabetes duration: no information Male = 29 (54 %) White = 28 (52 %)	Intervention Single educational intervention to provide families with basic diabetes management skills plus weekly phone calls for 3 months and then bimonthly. Study coordinator followed a standard protocol on the phone talking about problem-solving skills related to diabetes care. Duration of intervention: 6 months Control Standard care with quarterly visit with nurse practitioner and an endocrinologist	Intervention Baseline = 10 ± 1.4 % End-of-study = 9.5 ± 1.7 % Control Baseline = 10.2 ± 1.4 % End-of-study = 9.9 ± 1.6 % No significant reduction of HbA1c in intervention and control groups Adherence / Compliance evaluation: Adherence Clinician Checklist.
Weinger, 2011 [26]	*n* = 110^b^ Age = 46.6 years Diabetes duration = 23.7 years Male = 48 (48 %) White = 105 (96 %)	Intervention Five 2-hour sessions of manual-based group diabetes education Duration of intervention: 6 weeks Control Individual appointments with diabetes nurse and dietitian educators	Results of HbA1c were described together for patients with type 1 and type 2 diabetes Compliance evaluation of the educational intervention: frequency of diabetes self-care, 3-day pedometer readings, 24-hour diet recalls, average number of glucose checks
Nunn, 2006 [38]	*n* = 123 Age = 11.6 years Diabetes duration = 3.7 years Male = 69 (56 %) White = no information	Intervention Bimonthly phone calls from a diabetes educator covering the three main topics insulin use, carbohydrate intake and blood glucose values with a written educational program. Duration of intervention: 7 months Control Usual care	Intervention Baseline = 8.2 ± 1.1 % End-of-study = 8.9 ± 1.3 % Control Baseline = 8.3 ± 1.01 % End-of-study = 8.8 ± 1.1 % No significant reduction of HbA1c in intervention and control groups at 6 months Compliance evaluation of the educational intervention: SBGM, limited screen time, exercise practice, rotation of injection sites, warrant bracelets worn
Psychoeducation category			
Katz, 2014 [39]	*n* = 153 Diabetes duration = 12.8 years Male = 67 (44 %%) White = no information	Intervention 1 Psychoeducation was performed as 30-minute quarterly sessions with the patient, parent or guardian, and a non-medical care ambassador. Material was related to: family management of diabetes, problem-solving exercises and role-playing realistic expectations, glucose self-monitoring, avoidance of weight gain, and hypoglycemia. Duration of intervention: 2 years Intervention 2 Participants received monthly outreach by the care ambassador via phone or email, in addition to the quarterly diabetes care and ambassador care coordination. Duration of intervention: 2 years Intervention 3 Standard care including basic care coordination by the care ambassador (to assist in scheduling quarterly clinic visits)	Intervention 1 Baseline = 8.3 ± 1.4 % End-of-study = 8.6 ± 1.0 % Intervention 2 Baseline = 8.5 ± 1.4 % End-of-study = 8.8 ± 1.0 % Intervention 3 Baseline = 8.5 ± 1.4 % End-of-study = 8.6 ± 1.0 % No significant reduction of HbA1c in intervention and control groups at 2 years Compliance evaluation of the educational intervention: Diabetes Family Responsibility Questionnaire

^a^Results referred to two out of three study arms: behavior versus individual care; HbA1c results were from 73 patients

^b^Results referred to two out of three study arms: educational versus individual care; HbA1c results were from 73 patients

**Table 3:** Meta-analysis: risk of bias in individual randomized clinical trials evaluating interventions to improve compliance to lower glycated hemoglobin (HbA1c) in patients with type 1 diabetes according to intervention category

	Selection bias		Performance bias	Detection bias	Attrition bias	Reporting bias
	Random sequence generation	Allocation concealment	Blinding of participant and personnel	Blinding of outcome assessment	Incomplete outcome data	Selective reporting
Psychology category						
Ellis, 2005 –2007^b^	low	low	low	low	uncertain	low
Nansel, 2007	low	low	low	low	high	uncertain
Weinger, 2011^a^	low	low	low	low	uncertain	low
Nansel, 2011	low	low	high	uncertain	high	low
Mulvaney, 2010	low	low	uncertain	uncertain	uncertain	uncertain
Franklin, 2006	low	low	low	uncertain	uncertain	low
Telecare category						
Montori, 2004	low	low	low	uncertain	uncertain	low
Lawson, 2005	low	low	low	low	uncertain	low
Farmer, 2005	low	low	low	low	uncertain	low
Landau, 2011	low	low	low	low	uncertain	low
Gay, 2006	low	low	low	low	uncertain	low
Esmatjes, 2014	low	low	uncertain	uncertain	uncertain	low
Education category						
Cook, 2002	uncertain	uncertain	low	uncertain	uncertain	low
Howe, 2005	uncertain	uncertain	uncertain	low	uncertain	low
Weinger, 2011^b^	low	low	low	low	uncertain	low
Nunn, 2006	low	low	low	low	uncertain	low

^a^The same study had three arms evaluated as: psychology versus individual care and education versus individual care interventions

^b^Four published complementary reports

The data available from the reviewed RCTs allowed us to perform meta-analyses of psychological, educational, and telecare interventions. Only one trial evaluated combined psychological and educational interventions (psychoeducation category). Therefore, this trial was included only in the systematic review.

#### Psychological interventions

Systematic review of psychological interventions included six RCTs [21–29] and 783 patients aged 16.4 years (8 to 47), most of them, but one [26] conducted in children and adolescents. Patients were mostly white (75 %) and about half of them were male with a mean diabetes duration of 7 years (4 to 24) years. Baseline HbA1c in intervention was 9.2 % (8.7 to 11.4 %) and 9.1 % (8.2 to 11.3 %) in the control group. Duration of intervention was 10.2 months (11 weeks to 24 months). The psychological approaches used in RCTs are described in Table 1.

Nine studies were initially considered for inclusion in the psychological interventions meta-analysis [21–24, 26, 28]. However, the four studies conducted by Ellis et al. [21–24] presented complementary data and the same patients were evaluated. Then, we included data of only one study to avoid including the same individuals inappropriately twice in the pooled estimate. Therefore, four studies which presented baseline and end-of-study data were included in this meta-analysis.

The interventions promoted a significant reduction in HbA1c (MD −0.310 %; 95 % CI, −0.599, −0.021; *P* = 0.035). No heterogeneity was found in this analysis (*I*^*2*^ 0 %; *P* = 0.615).

#### Telecare interventions

Systematic review of telecare interventions evaluated six RCTs [30–35], including 494 patients with mean age 25.8 (13 to 43 years). No information about ethnicity was provided and about half of patients were males. Diabetes duration was 11.43 years (5.7–17.2). The length of most studies was 6 months and only one lasted for 9 months. The description of telecare interventions used in RCTs is shown in Table 1.

**Fig. 2:**
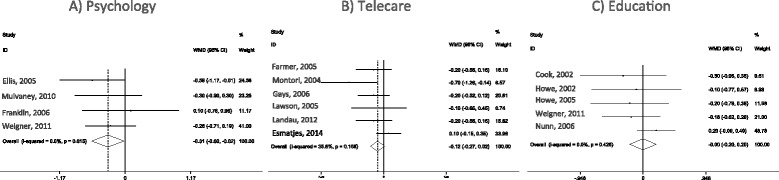
Forest plots of interventions to improve compliance with lower glycated hemoglobin (HbA1c) in patients with type 1 diabetes: **a** Psychological. **b** Telecare. **c** Education categories

All six RCTs were included in the meta-analysis of telecare intervention. The HbA1c of patients submitted to telecare interventions was not reduced during trials (MD −0.124 %; 95 % CI, −0.268, 0.020; *P* = 0.090) (Fig. 2). No heterogeneity was found in this analysis (*I*^*2*^ 35.8 %; *P* = 0.168).

### Educational interventions

Systematic review of educational interventions included four RCTs [26, 36–38] and 352 patients 19.6 years old (12 to 46). Trials were conducted mainly in children and adolescents. Patients were mostly white (65 %) and about half of them were male. Mean diabetes duration was described in only two studies. The duration of intervention varied from a single session to 12 months. The educational interventions used in RCTs are described in Table 1.

In the meta-analysis of included RCTs, five interventions were evaluated in four trials. No change in HbA1c was observed with educational approaches (MD −0.001 %; 95 % CI, −0.202, 0.200; *P* = 0.990) (Fig. 2). No heterogeneity was found in this analysis (*I*^*2*^ 0 %; *P* = 0.426).

#### Psychoeducation intervention

One trial combined psychological and educational intervention [39] including 153 patients with type 1 diabetes (44 % males, age 12.8 years) and evaluated three intervention arms. The psychoeducation arm consisted in 30-minute quarterly sessions. Psychoeducational material was related to family management of diabetes, avoiding perfectionism and setting realistic goals (psychological intervention), and glucose self-monitoring, weight gain, and hypoglycemia (educational intervention). Psychoeducational intervention was compared to usual care or usual care plus monthly phone calls or email reinforcements. Care from a non-medical ambassador occurred in all study arms. There was no difference in HbA1c among groups at 2 years.

#### Subgroup analyses

Twelve of the 18 meta-analyzed trials were conducted only in children and adolescents. We re-ran analyses maintaining only these 12 RCTs [21–24, 28, 29, 33–37]. Results of these meta-analyses are described in Table 4. No intervention (psychology, telecare, education) was able to reduce HbA1c in this age specific population. No heterogeneity was found in any meta-analysis.

**Table 4:** Subgroup meta-analyses: changes in glycated hemoglobin (HbA1c) (%) in randomized clinical trials evaluating interventions to improve compliance with lower HbA1c in children and teenagers with type 1 diabetes

Type of intervention	Number of studies	Number of patients	MD	95 % CI	*P*
			HbA1c		
Psychological [21–24, 28, 29]	3	239	-0.34%	-0.72 to 0.035 %	0.083
Telecare [32–35]	3	554	-0.18%	-0.40 to 0.03 %	0.098
Educational [36–38]	4	631	0.046	-0.80 to 0.272	0.689

*MD* mean differences

## Discussion

This was a systematic review of interventions aiming to reduce HbA1c in patients with type 1 diabetes by improving compliance with therapy. The review considered 1782 individuals from 19 RCTs. We were able to perform three meta-analyses according to the type of interventions: psychology, telecare, and education. Psychological interventions were associated with HbA1c reduction (MD −0.310; 95 % CI, −0.599 to −0.0210, *P* = 0.035) but not meta-analyses of telecare (MD −0.124 %; 95 % CI, −0.268, 0.020; *P* = 0.090) or educational (MD −0.001; 95 % CI, −0.202, 0.200; *P* = 0.990) interventions.

Tight glycemic control is difficult to obtain in type 1 diabetic patients and any intervention that reduces HbA1c is extremely helpful in controlling their diabetes. We identified two other systematic reviews on adherence in the medical literature [13, 14]. One of them analyzed cross-sectional studies [13]. The other study evaluated adherence or self-management promoting strategies. However, the authors performed a meta-analysis including all different categories of intervention together and showed no improvement in glycemic control [14]. We believe that stratifying intervention categories (such as psychology, education, telecare), as we did in the current study, is a more adequate statistical approach. In addition, it is important to emphasize that our literature search method was quite unique: we performed an open search for RCTs that improved patient compliance instead of searching for specific interventions. This strategy could explain the differences between our results and the previous reviews. We also excluded crossover trials because it is hard to perform an adequate washout when dealing with subjective interventions.

A systemic review of psychological and educational interventions in adolescents with type 1 diabetes [40] seemed to reduce HbA1c for both interventions but the confidence interval for HbA1c changes was quite large. Furthermore, this review was not projected to evaluate whether the studied interventions were associated with patients’ compliance with the diabetes treatment. Regarding telecare intervention, a meta-analysis conducted in patients with type 1 diabetes did not reduce HbA1c [30], similar to our results. However, that study was not aimed to reduced HbA1c [30], and once again this study was not designed to evaluate compliance.

Most children and teenagers with type 1 diabetes do not meet traditional glycemic control targets [11] and recently the American Diabetes Association reduced HbA1c goals for youth [12]. Interestingly, in our data search we found that most studies were performed in children and adolescents. Therefore, we decided to perform a subgroup analysis including only these patients. Indeed, the management of children and teenagers with diabetes usually has peculiarities, including non-pharmacological interventions (e.g., family involvement) [18]. Unfortunately, we were not able to confirm benefits of any studied intervention in this specific population similar to those described by other authors [40]. Inclusion of a greater number of studies in pediatric patients could have shown improvement in glycemic control since there is still a clear trend to lower HbA1c by psychological intervention according to our subgroup analysis.

A possible limitation of our systematic review could be the small number of studies in each intervention category. Since we performed complete-case analyses [48], the missing data in some of reviewed trials precluded their inclusion in our meta-analyses. In theory the choice to use only studies that report baseline and final values could lead to the possibility of selective reporting [43]. Indeed, analyses based on changes from baseline will be more efficient and powerful than comparisons of final values [43], especially when analyzing HbA1c values. The quality of included studies could represent a weakness in our meta-analyses. Nevertheless, only the study of Nansel et al. [27] included a psychological intervention and there were two high domain biases: blinding bias, which was not truly applicable to this type of intervention, and incomplete data. All other studies included revealed a low or uncertain bias risk. Contact with non-responding authors remains a problem in performing meta-analyses since we could not recover any missing data after personal contact. It would be interesting to compare all included trials through a network meta-analysis. However, different strategies without a common comparator prevented us from performing this analysis.

## Conclusion

We performed this systematic review because there was no clear information available regarding which kind of intervention should be used to improve compliance with general diabetes treatment aimed at lowering HbA1c (improvement of glycemic control). Unfortunately, so far we could only demonstrate psychological intervention as the sole evidence-based recommendation; the number of included studies was relatively low but their quality allowed us to conclude that this tool can be useful in the management of diabetic patients. In conclusion, we demonstrated that in patients with type 1 diabetes psychological interventions to improve patients’ compliance with diabetes treatment did improve glycemic control.

## Appendix

### Research strategy

#### Medline

((((“Randomized Controlled Trial”[Publication Type]) AND “Diabetes Mellitus, Type 1”[Mesh]) AND “Patient Compliance”[Mesh]) AND “Hemoglobin A, Glycated”[Mesh])

## Additional file

Additional file 1:
**PRISMA 2009 Checklist.** (DOC 63 kb)

## Abbreviations

HbA1c: glycated hemoglobin
MD: mean differences
PRISMA: Preferred Reporting Items for Systematic Reviews and Meta-Analyses
RCT: randomized controlled clinical trial
